# Primary auditory cortex representation of fear‐conditioned musical sounds

**DOI:** 10.1002/hbm.24846

**Published:** 2019-10-30

**Authors:** Matthias Staib, Aslan Abivardi, Dominik R. Bach

**Affiliations:** ^1^ Computational Psychiatry Research, Department of Psychiatry, Psychotherapy, and Psychosomatics Psychiatric Hospital, 8032 University of Zurich Zurich Switzerland; ^2^ Neuroscience Center Zurich 8057 University of Zurich Zurich Switzerland; ^3^ Wellcome Centre for Human Neuroimaging University College London London UK

**Keywords:** associative learning, discriminative fear conditioning, emotional learning, multivariate pattern analysis, spectrotemporal information, threat conditioning, threat representation

## Abstract

Auditory cortex is required for discriminative fear conditioning beyond the classical amygdala microcircuit, but its precise role is unknown. It has previously been suggested that Heschl's gyrus, which includes primary auditory cortex (A1), but also other auditory areas, encodes threat predictions during presentation of conditioned stimuli (CS) consisting of monophones, or frequency sweeps. The latter resemble natural prosody and contain discriminative spectro‐temporal information. Here, we use functional magnetic resonance imaging (fMRI) in humans to address CS encoding in A1 for stimuli that contain only spectral but no temporal discriminative information. Two musical chords (complex) or two monophone tones (simple) were presented in a signaled reinforcement context (reinforced CS+ and nonreinforced CS−), or in a different context without reinforcement (neutral sounds, NS1 and NS2), with an incidental sound detection task. CS/US association encoding was quantified by the increased discriminability of BOLD patterns evoked by CS+/CS−, compared to NS pairs with similar physical stimulus differences and task demands. A1 was defined on a single‐participant level and based on individual anatomy. We find that in A1, discriminability of CS+/CS− was higher than for NS1/NS2. This representation of unconditioned stimulus (US) prediction was of comparable magnitude for both types of sounds. We did not observe such encoding outside A1. Different from frequency sweeps investigated previously, musical chords did not share representations of US prediction with monophone sounds. To summarize, our findings suggest decodable representation of US predictions in A1, for various types of CS, including musical chords that contain no temporal discriminative information.

## 1 INTRODUCTION

After repeated coupling of neutral cues (conditioned stimuli, CS) with an aversive event (unconditioned stimulus, US), an associative link between the two stimuli is formed in a process termed fear conditioning (or threat conditioning). As a result, the conditioned stimulus (CS) is predictive for the US and elicits a conditioned fear response. In nonhuman animals, there is a body of evidence that this type of learning requires synaptic plasticity in at least two amygdala nuclei (Herry & Johansen, 2014). In addition, auditory cortex (ACX) is crucially required for fear conditioning when an animal has to discriminate CS+ and CS− (Teich et al., 1988), indicating that ACX may enable the inhibition of responding to the CS−. When CS+ differs from CS− in its spectro‐temporal features (Ohl, Wetzel, Wagner, Rech, & Scheich, 1999), ACX is required to facilitate responding to the CS+ during acquisition. These studies demonstrate a differential involvement of ACX in acquiring CS+ and CS− responding, possibly based on a disinhibitory mechanism (Letzkus et al., 2011). For complex CS+ conditioning, disinhibitory input from the primary auditory cortex (A1) to amygdala during and shortly after US presentation is suggested to be part of the fear‐conditioning macrocircuit (Letzkus et al., 2011; Weible, Liu, Niell, & Wehr, 2014). In these two studies, a frequency modulated sound (Letzkus et al., 2011) or a silent gap in a continuous sound (Weible et al., 2014) served as a CS that co‐terminated with the US. When A1 was optogenetically inhibited during US presentation, learning was disrupted.

Another line of evidence for ACX involvement in fear conditioning comes from postconsolidation observations. After fear memory is consolidated (within 24 hr), a wealth of evidence indicates plastic reconfiguration of ACX (Weinberger, 2007). For example, individual neurons responded more to the CS+ frequency after fear memory consolidation than before training, and the entire tuning curve shifted toward CS+ frequency (Bakin & Weinberger, 1990). Furthermore, ablation of ACX after consolidation resulted in reduced fear responses while lesions before learning did not impair fear acquisition (Romanski and LeDoux, 1992). This indicates that even for simple nondiscriminative fear learning, ACX is involved and becomes necessary for expression of fear memory after consolidation (Romanski et al., 1993), although it is not crucially required during acquisition.

Bringing these findings together, it appears plausible that the ACX should be involved in stimulus discrimination already during CS presentation, and send such information to the amygdala at the time point of the US. Yet, until today it is unclear what exactly the ACX encodes during CS presentation.

We have previously used multivariate functional magnetic resonance imaging (fMRI) during discriminative fear conditioning in humans to demonstrate encoding of US prediction, before US onset, in Heschl's gyrus (Staib & Bach, 2018). This may indicate that A1 not only extracts stimulus features and transmits this information to the amygdala together with the US, but that it already computes the US prediction during CS presentation. These results were unlikely to be caused by postconsolidation synaptic plasticity since the experiment was too short to include such changes. Crucially, this representation of US predictions was found for simple monophone sine tone CS, and for frequency sweep CS. For these particular two types of sounds, the representation of US prediction was shared: fMRI patterns relating to CS+ and CS− in Heschl's gyrus were the same for both types of sounds, that is, they could be cross‐decoded (Staib & Bach, 2018).

Notably, these frequency sweeps were modeled after rodent experiments. For rodents, such sounds are part of species‐specific vocalization (Ohl et al., 1999), and similar frequency changes are also common in human prosody. Here, CS+ and CS were discriminated by complex spectro‐temporal information. We sought to extend these previous findings to sounds only discriminated by spectral but not temporal features. To this end, we chose a class of artificial sounds that do not form part of our natural sound environment, but which most humans are familiar with, namely musical chords (here triads composed of 3 sine tones). Together with our previous findings, this approach allows investigating a differential involvement of A1 in processing spectral and/or temporal features. Furthermore, our previous finding was based on a group‐level representation of Heschl's gyrus. This group‐level definition provided only an imprecise approximation to individual Heschl's gyrus’ boundaries, due to high interindividual variability in gyrification patterns (Leonard, Puranik, Kuldau, & Lombardino, 1998; Marie et al., 2015). Furthermore, within Heschl's gyrus there is heterogeneity in A1 location, which in case of duplication is exclusively situated in the most anterior gyrus (Brodmann, 1909; Galaburda & Sanides, 1980; Shapleske, Rossell, Woodruff, & David, 1999). Here, we used a more precise, participant‐level definition of A1, by capitalizing on the shape of Heschl's gyrus in individual segmentation of MRI images. Thus, we investigated whether fMRI patterns in A1 discriminated between CS+ and CS− to a greater extent than they discriminated between similar pairs of neutral sounds (NS) that were explicitly never coupled with a US, under identical task demands. Furthermore, we sought to confirm that the detectability of this encoding was not different between simple monophone sine tones and complex triads. Finally, we sought to examine whether the patterns representing the US prediction were shared between simple and complex sounds, that is, whether they were stimulus‐invariant and cross‐decodable between the two types of sounds, as we have previously shown for monophones and frequency sweeps (Staib & Bach, 2018).

## 2 MATERIALS AND METHODS

### 2.1 Participants and design

From the general and student population, we recruited 20 healthy, right‐handed participants (10 female, mean age: 24.8 years, age range 19–35) for the fMRI experiment. The fMRI experiment was preceded by a behavioral control experiment to ensure that the musical sounds were associable with an aversive US to the same extent as the monophone sounds. To this end, we recruited 22 different participants (15 female, mean age: 22.3, age range 18–32). The size of the fMRI sample was based on a power analysis using Gpower (Erdfelder, Faul, & Buchner, 1998) and data from our previous experiment (Staib & Bach, 2018). For the effect of context on CS or NS discriminability, we had observed partial η^2^ = 0.153 (resulting in an effect size of *f* = 0.426) and a correlation between measurements in the two contexts of *r* = .25. Thus, to detect an effect of context on stimulus discriminability with 95% power and at an alpha level of .05 in a repeated‐measures ANOVA, a sample size of *N* = 20 was required.

Because discriminant fear conditioning requires sensory discrimination of the sounds, which we suspected could vary between individuals, we included participants into the fMRI sample only if they met a minimum requirement in a preceding sound discrimination task (see details in task procedure). The experiment and the form of taking written informed consent were in accordance with the Declaration of Helsinki and approved by the governmental ethics committee (KEK‐ZH 2013‐0258). Both experiments used a 3‐way factorial design with the within‐subjects factors stimulus (S1, S2), complexity (simple, complex) and context (reinforcement, instructed nonreinforcement).

### 2.2 Stimuli

**Figure 1:**
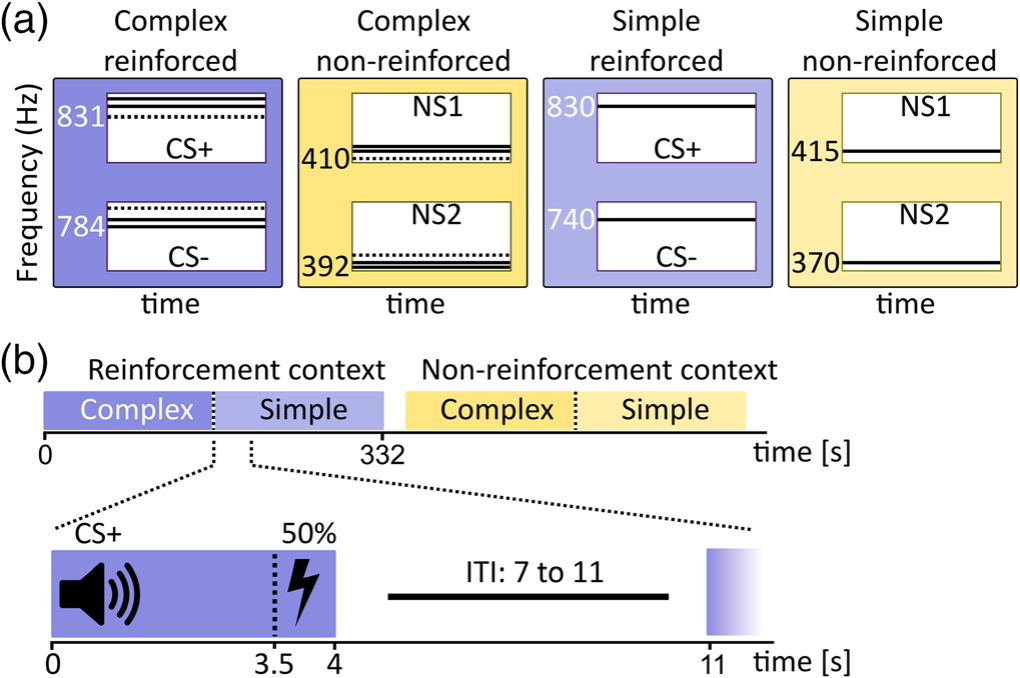
(a) We compared “simple” monophone sounds with “complex” triads, in two different contexts: A reinforcement context with CS+ (reinforced) and CS− (nonreinforced), and a nonreinforcement context with neutral sounds (NS) in which participants were explicitly instructed about the absence of the US. Frequencies are shown for each bass tone. Dashed lines signify the root of each triad that served as discriminative spectral feature between two chords. (b) Block order in the fMRI experiment, and intra‐trial procedure

Sounds were 4 s long and either monophone sine sounds (simple) or the combination of three simultaneous monophone sine sounds (complex, Figure 1). CS and NS were one octave apart. The assignment of CS/NS to octave was balanced across participants. Simple sounds had time‐invariant frequencies of 370 and 415 Hz, or 740 and 830 Hz. Each complex sound was a major chord of three sounds, that is, a triad. The root of the triad, that is, the note that defines its musical quality, was the discriminative feature, while the highest note was kept constant. The triad was either in root position or in first inversion. The frequencies were then slightly adapted such that the overall pitch of pairs of chords was perceived as similar in the MR scanner, rendering the root as the discriminative feature between two chords. The triads were composed of [392.0 Hz, 466.2 Hz, 622.3 Hz] and [410.0 Hz, 516.6 Hz, 614.3 Hz], or [784.0 Hz, 932.3 Hz, 1,244.5 Hz] and [830.6 Hz, 1,046.5 Hz, 1,244.5 Hz], respectively. For the qualifier task prior to the MRI experiment, different chords with frequencies of [220.0 Hz, 261.6 Hz, 349.2 Hz] and [225.0 Hz, 283.5 Hz, 337.1 Hz] were used. Details of sound creation, sound volume adjustment, and sound delivery matched those described in Staib and Bach (2018).

US were applied for 500 ms and consisted of 3 square electric pulses to the forearm of 200 μs duration and 100 ms onset asynchrony (pin‐cathode/ring‐anode, Digitimer DS7A, Digitimer, Welwyn Garden City, UK). Stimulation current was determined in two phases: (1) staircase testing phase, to determine the maximum current tolerated by the participant; (2) random testing phase with 14 US intensities that were rated on a scale from 0 (not perceived) to 100 (clearly painful). Phase (2) was repeated immediately after the experiment. Final US current (mean ± SD: 7.40 ± 1.38 mA) was clearly uncomfortable but not painful (rating before entering the scanner: Mean ± *SD*: 85.6 ± 11.9; rating after leaving the scanner: Mean ± *SD*: 81.6 ± 22.6). Participants in the fMRI experiments reported US habituation (excluding one subject due to loss of the electrode at the end of measurement), *t*_18_ = 4.83, *p* < .001. In the behavioral control experiment, five participants did not complete the re‐evaluation. The remaining 17 participants showed no habituation or sensitization to the US, *t*_16_ = 1.00, *p* = .33.

Finally, reinforcement and nonreinforcement contexts were signaled by yellow or blue screen background, with assignment balanced across participants.

### 2.3 Task procedure

For inclusion into the fMRI experiment, we required at least 75% accuracy in a sound discrimination task (qualifier) presented via headphones outside the MRI and using sounds similar to the ones used during fMRI. After a short training of five repetitions, participants were asked to press the correct button in at least 15 out of 20 repetitions.

Next, in a training session without reinforcement, simple tones and their corresponding complex tones were introduced. Each sound was played twice and accompanied by a visual description, together with the instruction which button to press for each sound. The same sounds served as CS or NS during the subsequent acquisition phase.

In the main task, CS+ was only reinforced in a reinforcement context, signaled by background color. In the nonreinforcement context, different sounds were presented. We instructed participants that US would never occur in the nonreinforcement context, while in reinforcement context, US probability depended on the type of CS but not on their behavior.

The experiment was broken up into 8 blocks with average duration of 332 s, separated by short breaks. Each block of 24 trials took place in either reinforcement or nonreinforcement context, in alternating order (Figure 1b). The first 12 trials of each block were simple sounds, and the second 12 trials complex sounds, or vice versa, balanced across participants. Within these 12 trials, trial order was randomly permuted, and the same order was used for all participants. Inter‐trial interval was randomly drawn from {7 s, 9 s, 11 s}. Across the entire experiment, each of the eight stimuli was presented 24 times. The precise trial sequence, and the possible inter‐trial intervals, maximized the variance for the contrast CS+/CS− in the resulting design matrix, thus optimizing power for the analysis of the BOLD signal (Ulmer & Jansen, 2010).

Participants were instructed to indicate the identity of each sound with a key press, as trained beforehand. They received feedback (change in fixation cross color) at the end of the CS/NS if they pressed a wrong button or did not respond within 3 s of CS/NS onset. In the fMRI experiment, accuracy (hit rate) averaged per participant was between 87% and 99%. As a manipulation check, accuracy per condition was analyzed in a generalized linear mixed effects model with Satterthwaite approximation to degrees of freedom (Luke, 2017). This revealed that participants responded more accurately to CS+ and to those NS that required the same key press (mean ± SEM 97.07% ± 3.77%) than to CS− and the other NS (95.28% ± 4.74%, *F*_1, 3,795_ = 5.79; *p* = .016). They also responded more accurately to simple (96.86% ± 4.63%) than to complex stimuli (95.49% ± 3.90%, *F*_1, 3,795_ = 4.00; *p* = .046). Context had no effect on accuracy, and there were no interactions. In a linear mixed‐effects model with Satterthwaite approximation to degrees of freedom (Luke, 2017), reaction times (Table 1) were shorter during reinforcement than nonreinforcement context (*F*_1, 3,796_ = 10.48; *p* = .001) and shorter for CS− and response‐matched NS than CS+ and response‐matched NS sounds (*F*_1, 3,796_ = 43.42; *p* < .001). This was particularly pronounced in the reinforcement context (interaction CS × context: *F*_1, 3,796_ = 8.86; *p* = .003). The fact that responses were also slower for CS+ response matched NS than CS− response matched NS may indicate some level of fear generalization via the associated motor response. Also, the CS+/CS− difference was more pronounced for simple sounds (interaction CS × complexity: *F*_1, 3,796_ = 15.64; *p* < .001).

**Table 1:** Reaction time statistics

Marginal means (SEM) in ms	CS−	CS+	NS1	NS2
Simple	737 (36)	852 (28)	782 (37)	868 (43)
Complex	776 (40)	844 (48)	850 (47)	832 (48)

*Note:* Participants were instructed to respond quickly, within a response time limit of 3 s.

### 2.4 Skin conductance data acquisition

We recorded skin conductance on left thenar/hypothenar (MRI experiment: Biopac MP150/ GSR‐100C; behavioral control experiment: LabLinc V71‐23, Coulbourn; DI‐149/Windaq, Dataq) as described previously (Staib & Bach, 2018). Sampling rate was 1,000 Hz in the MRI experiment and 200 Hz in the behavioral control experiment.

### 2.5 MRI data acquisition

Data was recorded on a 3 T (Philips Achieva, Best, The Netherlands) whole‐body MRI scanner. We acquired two high‐resolution T1‐weighted scans (field of view, 255 × 255 × 180 mm; matrix, 336 × 334; 237 sagittal slices [0.77 mm]). Functional images were acquired using a 1.5 mm isotropic T2*‐weighted echo‐planar (EPI) sequence (TR, 2.5 s; echo time, 30 ms; flip angle, 85°; in‐plane field of view, 216 × 216 mm; matrix, 144 × 144; slice thickness, 1.5 mm; interleaved slices; slice tilt 30°) with partial brain coverage including the entire auditory cortex. Field of view was centered on ACX. We had no specific hypotheses about CS representation in the amygdala, and due to a technical oversight, slice tilt/phase‐encoding gradient polarity was not optimized to reduce susceptibility artifacts in this region. This is why our analysis focuses on A1, the primary region‐of‐interest.

### 2.6 MRI data analysis

We preprocessed MR images with the default procedures in SPM12 (Wellcome Centre for Human Neuroimaging, London, UK). Using a B0 scan for each participant, the SPM12 FieldMap toolbox, and the realign & unwarp procedure, we corrected all BOLD images for static distortions due to field inhomogeneities, for changes in these distortions due to head motion, and for head motion itself (Andersson, Hutton, Ashburner, Turner, & Friston, 2001; Hutton et al., 2002). After slice time correction (Sladky et al., 2011), we coregistered BOLD images to the native‐space T1 image.

To estimate trial‐by‐trial amplitude of the BOLD response to auditory stimuli in a general linear model (GLM), we constructed a design matrix that contained one regressor per trial. To this end, a stick function for each event was convolved with the default canonical hemodynamic response function. This procedure appropriately estimates the trial‐by‐trial BOLD amplitude at the given inter‐trial‐interval (Mumford, Turner, Ashby, & Poldrack, 2012). The design matrix contained one further regressor per run for the US, constructed with a stick function across all trials and convolved with the hemodynamic response function, as well as a standard high‐pass filter. We did not analyze estimated BOLD responses from trials that were reinforced with the US (Bach, Weiskopf, & Dolan, 2011; Staib & Bach, 2018), to avoid contamination by residual motion artifacts due to the US.

For mass‐univariate and searchlight analysis, we used the unified segmentation procedure in SPM (Ashburner and Friston, 2005) to estimate deformation parameters that map MNI space to each participant's native space.

### 2.7 Region of interest definition

Automated parcellation of cortical structures on T1‐weighted images was performed in native space using the “recon‐all” pipeline in FreeSurfer Version 6.0 (http://surfer.nmr.mgh.harvard.edu/; Dale, Fischl, & Sereno, 1999; Fischl et al., 2002; Fischl et al., 2008; Fischl, Liu, & Dale, 2001; Fischl, Sereno, & Dale, 1999; Fischl, Sereno, Tootell, & Dale, 1999; Segonne, Grimson, & Fischl, 2005). In this approach, anatomical labels are assigned by combining local geometric information and atlas data acquired from a manually segmented training set (Desikan et al., 2006; Destrieux, Fischl, Dale, & Halgren, 2010; Fischl et al., 2004). The anterior transverse temporal gyrus (of Heschl) parcellation (“G_temp_sup‐G_T_transv”) as described in Destrieux et al. (2010) was extracted. The FreeSurfer labeling takes care of the high interindividual variability and frequent duplications of the transverse temporal gyrus (Leonard et al., 1998; Marie et al., 2015). It thus separates the most anterior gyrus which exclusively contains A1 (Shapleske et al., 1999). Nevertheless, partial duplications and to lesser extent complete duplications of the transverse temporal gyrus, often remained undetected by the algorithm and hence were removed by manually defining coordinates for a separating plane using MATLAB. We note that on the lateral axis, the granular core field A1 is mainly located in the medial two‐thirds of Heschl's gyrus according to cytoarchitecture (Hackett, Preuss, & Kaas, 2001; von Economo & Koskinas, 1925; Wallace, Johnston, & Palmer, 2002). Our parcellation includes the entire gyrus and thus may possibly extend into other subregions (e.g., primary subfields R/RT or lateral belt). Tonotopic mapping of ACX using high‐resolution fMRI has been suggested as an alternative localization technique of subfields (Da Costa et al., 2011; Leaver & Rauschecker, 2016) revealing further organizational complexity as well as variability in A1 location.

### 2.8 Multivariate image analysis

BOLD response estimates from bilateral A1 were extracted from the GLM output images with spm_searchlight. We then sought to discriminate the two stimuli in each of four task conditions (simple CS, complex CS, simple NS, complex NS) with a support vector machine (SVM; Chang and Lin, 2011) following a threefold cross‐validation scheme established in our previous work (Staib & Bach, 2018).

Independently for each voxel, we *z*‐scored BOLD response estimates across all trials, in order to avoid numerical instability. For each task condition, there were 48 stimuli: 24 CS− or NS1, and 24 CS+ or NS2. Of the 24 CS+, 12 were reinforced and thus discarded from the analysis. For the reinforcement context, we thus trained a SVM on 24 CS (16 CS−, 8 CS+) per complexity condition and tested it on the remaining 12 CS (8 CS+, 4 CS−), for each of the three folds. Because CS representations presumably change over time during learning, we did so by creating a running index over each individual stimulus and including every third stimulus into the test data set such that both training and test data were drawn from the entire duration of experiment. Because of the different numbers of CS− and unreinforced CS+ in the reinforced context, a binomial statistical test is not appropriate. We estimated the SVM performance under the null hypothesis of label exchangeability. To this end, we randomly permuted stimulus labels 1,000 times and repeated the classification procedure (Bach et al., 2011; Staib & Bach, 2018). The average classification accuracy with permuted labels (chance level) was 59.9%, with a standard deviation of 6.7% across participants, ROIs, and conditions. Averaging over all random permutations, we could thus subtract, for each task condition, expected classification accuracy under the null hypothesis from the classification accuracy with correct labels. For the nonreinforcement context, different from CS, all 48 trials per condition could potentially be used for fMRI analysis, since there was no US. To render CS and NS analysis analogous in terms of the number of trials in the SVM, we removed 12 random trials from one randomly selected NS (NS1 or NS2) before MVPA. We averaged over 100 repetitions of this procedure per permutation of the trial labels.

Cross‐prediction was performed to investigate stimulus invariance of CS representations (i.e., to interrogate whether monophone CS+/CS− were discriminated by the same patterns as the triad CS+/CS−). For cross‐prediction, we used all simple CS or complex CS as training data set, and all CS from the other category as test data set. We did the same procedure for NS, where NS identities were matched between simple and complex by the required key press, to rule out that CS cross‐prediction is due to motor responses.

Our main outcome measures are the difference in information content between two conditions, and cross‐prediction performance. The true value for both of these values can be above or below zero (Staib & Bach, 2018). In contradistinction, the absolute value of information encoding cannot be negative, which motivates scrutinizing the use of standard statistical tests in this situation (Allefeld et al., 2016). However, this is not an issue here.

To investigate threat encoding outside A1, we used a searchlight with 10 mm diameter in the function spm_searchlight (Kriegeskorte et al., 2006). Individual results were mapped to MNI spaces and analyzed with one‐sample t‐tests across the group.

### 2.9 Behavioral analysis

We estimated anticipatory sympathetic arousal after each CS or NS (Bach et al., 2010a; Staib, Castegnetti, & Bach, 2015) with PsPM 3.0 (http://pspm.sourceforge.net/; Bach et al., 2018), using the canonical skin conductance response function (Bach et al., 2010b). We used standard settings optimized for fear learning (Staib et al., 2015). Estimated sympathetic arousal for each participant was *z*‐scored across all trials (Staib et al., 2015; Staib & Bach, 2018)

### 2.10 Inference statistics

For fMRI analysis, BOLD discriminability for each pair of CS or NS was computed and analyzed in a complexity × context factorial model. For behavioral analysis, levels of the stimulus factor were defined by the required response, and data were analyzed in a stimulus × complexity × context factorial model.

Inference statistics were done in R 3.4.3 (http://www.r-project.org) using linear mixed‐effects models (lme4; Pinheiro & Bates, 2006). Degrees of freedom were estimated using the Sattertwaithe approximation (lmerTest; Luke, 2017). Fixed factors were CS, context, and complexity for sympathetic arousal, as well as context, complexity and hemisphere for MVPA classification results, and context and direction of cross‐prediction for cross‐classification results. We modeled a random intercept for each subject. For post hoc tests on smaller number of data points, we used Wilcoxon tests.

Mass‐univariate results and searchlight‐results were performed in SPM. Group‐space images were smoothed with an 8 mm FWHM Gaussian kernel. For mass‐univariate analysis, we examined the planned contrasts (CS+ > CS−) > (NS1 > NS2) [analogous to the main effect of context in MVPA], (CS+ > CS−) > (NS1 > NS2) complex, (CS+ > CS−) > (NS1 > NS2) simple, (CS+ > CS−), (CS+ > CS−) complex, (CS+ > CS−) simple, (NS1 > NS2), (NS1 > NS2) complex, and (NS1 > NS2) simple. As a plausibility check, we furthermore report the contrast CS versus baseline and US/CS+ versus no US/CS+. We corrected for family‐wise error with a random field theory approach, a voxel‐inclusion threshold of *p* < .001, and a reporting threshold of *p* < .05 (Worsley et al., 1992). The random field theory‐based approach appropriately controls the false positive rate when used with these *p* values (Eklund et al., 2016).

## 3 RESULTS

### 3.1 Fear acquisition is similar for complex and simple sounds

**Figure 2:**
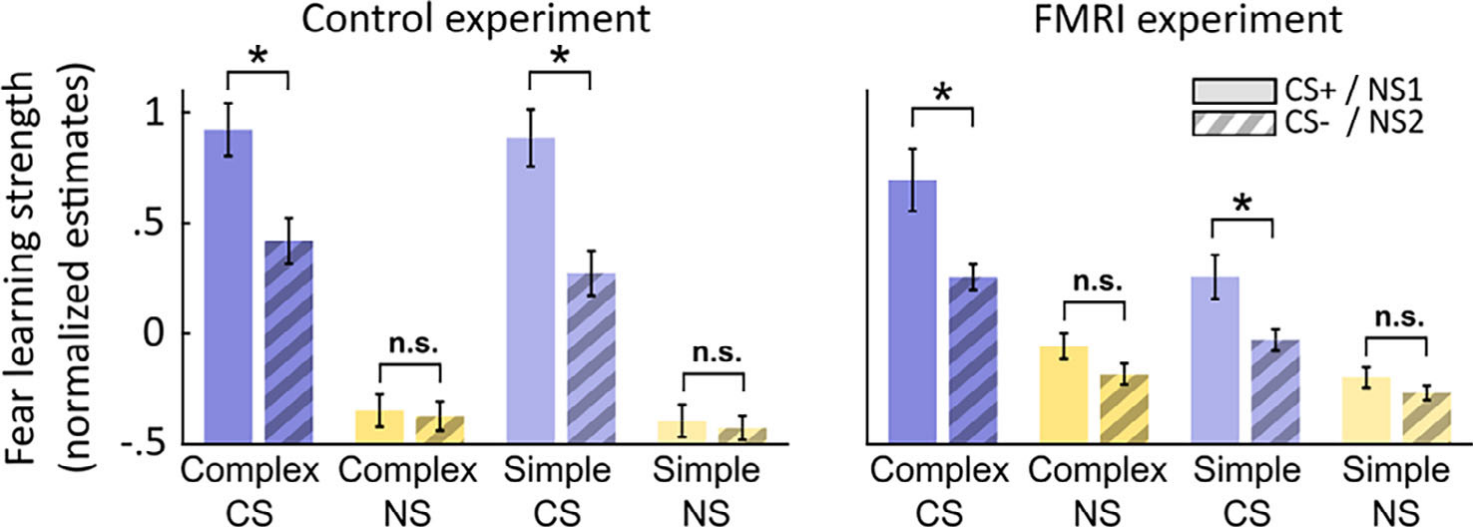
Fear learning strength, quantified as CS/NS‐associated sympathetic arousal, that is, amplitude of estimated central input into the sudomotor/sweat gland system, measured by skin conductance responses. Error bars: Group‐level SEM

Fear learning occurred in the reinforcement context (CS × context interaction) in both experiments (Figure 2, Table 2). There was no three‐way interaction with complexity, indicating similar learning for both simple and complex stimuli. In the reinforcement context, fear learning could be demonstrated for simple (*t*_21_ = 3.8, *p* < .001) and for complex sounds (*t*_21_ = 3.6, *p* = .002) in the behavioral sample, as well as in the fMRI sample (simple: *t*_19_ = 2.5, *p* = .02; complex: *t*_19_ = 2.9, *p* = .008). Furthermore, in both experiments, stimulus‐associated arousal was overall stronger in the reinforcement context than in the nonreinforcement context (main effect context) and for complex than for simple sounds (main effect complexity). In the fMRI experiment, arousal during complex sound presentation was higher than for simple sounds only in the reinforcement context (interaction context × complexity).

**Table 2:** ANOVA on CS/NS‐associated sympathetic arousal

	Behavioral experiment			fMRI experiment		
	*df*	*F*	*p*	*df*	*F*	*p*
CS	1, 3,261	84.1	<.001	1, 3,352	43.8	<.001
Context	1, 3,261	997.1	<.001	1, 3,352	183.9	<.001
Complexity	1, 3,261	5.0	.026	1, 3,352	46.6	<.001
CS × context	1, 3,261	68.4	<.001	1, 3,352	14.4	<.001
CS × complexity	1, 3,261	<1	.37	1, 3,352	2.3	.12
Context × complexity	1, 3,261	<1	.51	1, 3,352	12.7	<.001
CS × context × complexity	1, 3,261	<1	.43	1, 3,352	<1	.48

*Note:* The CS factor has levels CS+/response matched NS, and CS−/response‐matched NS. Results demonstrate similar fear learning (main effect CS and CS × context interaction) in both complexity conditions.

### 3.2 Mass‐univariate effects of CS and US

**Figure 3:**
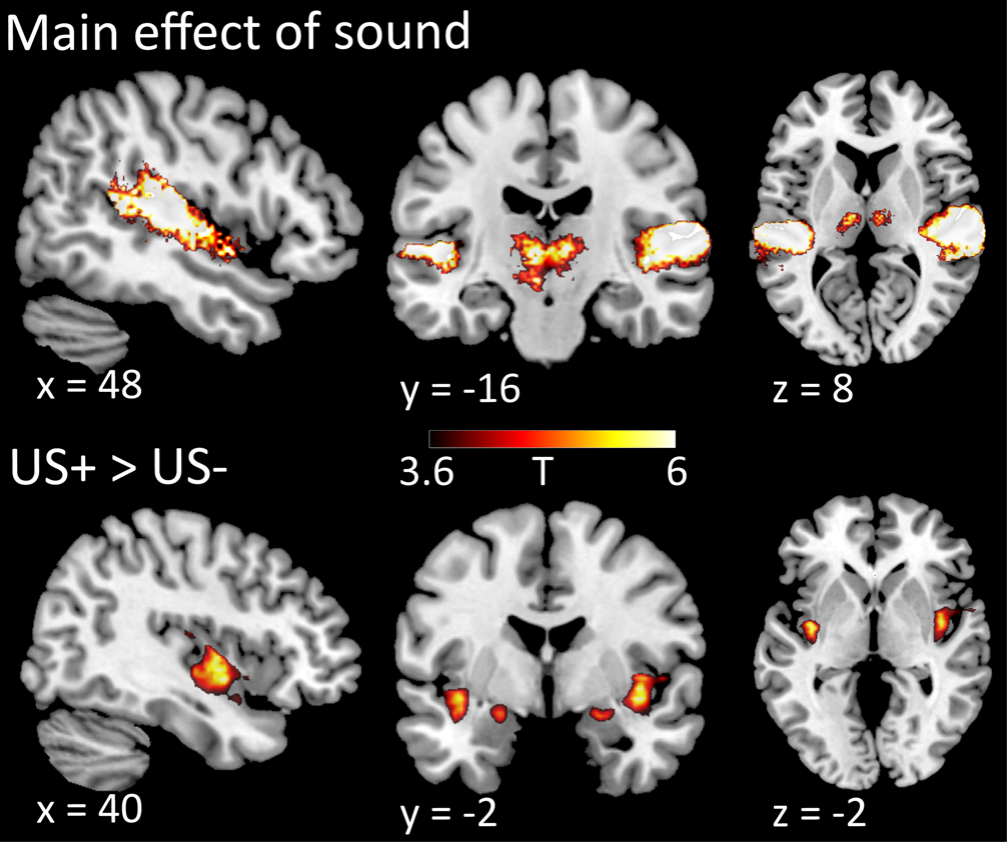
Mass‐univariate contrast of all stimuli versus baseline (main effect of sound) and US+ > US− after CS+ (*p* < .05 FWE‐corrected). Within the field of view, sounds evoke BOLD signal across temporal plane and superior temporal gyrus as well as in thalamic structures, and US evoke BOLD signal in insula and amygdala

In a contrast of all sounds against baseline, we observed BOLD responses across the entire ACX and in the thalamus (Figure 3). The US, compared to omission of US, activated a region within the insula and amygdala. We observed no significant effects of stimulus, context, or complexity, or any interaction in the mass‐univariate analysis, that is, all *p* > .05 FWE‐corrected on cluster level and peak level across the whole brain, consistent with previous work (Staib & Bach, 2018). In particular, we found no indication of global auditory attention in terms of increased BOLD signal for the CS+, or for stimuli in the reinforcement as opposed to nonreinforcement context. Also, no significant mass‐univariate results were found in a ROI analysis in A1.

### 3.3 Threat representation in A1

**Figure 4:**
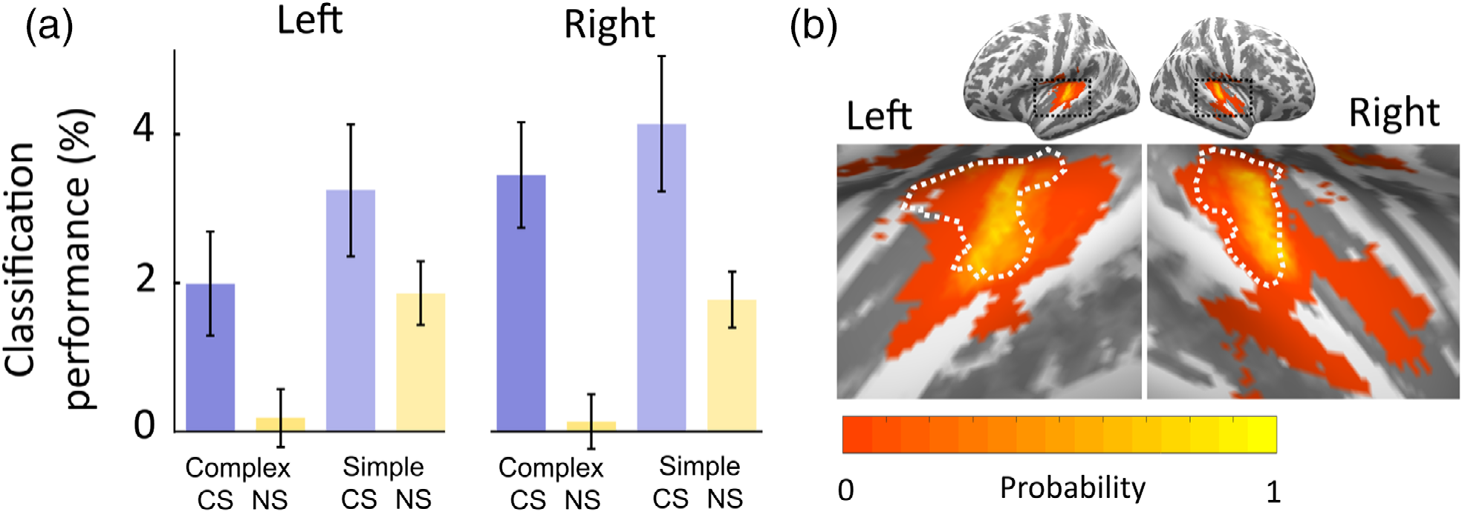
(a) Discriminability (mean ± between‐participant SEM above baseline performance estimated in a random permutation test) of multivoxel BOLD patterns to CS+/CS− or NS1/NS2 within A1. CS is better distinguished than NS across simple (monophone) and complex (triads) sounds. (b) Region of interest definition within Heschl's gyrus: Probability map of MNI‐normalized mask across participants, projected onto flattened cortex template. White dashed boundaries outline the atlas‐based mask of Heschl's gyrus for comparison

Confirming our primary hypothesis, classification of CS+/CS− was significantly higher than for NS (main effect of context) in A1 (Figure 4, Table 3). Because the physical stimulus difference between the NS pairs is comparable to the CS pairs, this suggests that the higher CS classification accuracy reflects a representation of the CS–US association, or threat anticipation, of the CS. Simple sounds could be classified better than complex sounds (main effect complexity), but notably, and as in our previous work, CS discriminability was comparable for both types of sounds, that is, there was no complexity × context interaction.

**Table 3:** ANOVA results for classification of CS+/CS−

	A1			Heschl's gyrus		
Effect	*df*	*F*	*p*	*df*	*F*	*p*
Context	1, 119	30.9	<.001	1, 133	6.2	.014
Complexity	1, 119	10.8	.001	1, 133	10.6	.001
Hemisphere	1, 119	1.9	.17	1, 133	<1	.42
Context × complexity	1, 119	<1	.39	1, 133	1.5	.29
Context × hemisphere	1, 119	2.4	.12	1, 133	<1	.72
Complexity × hemisphere	1, 119	<1	.70	1, 133	<1	.69
Context × complexity × hemisphere	1, 119	<1	.73	1, 133	<1	.30

*Note:* A1: native‐space definition of A1 from individual anatomy. Heschl's gyrus: Probabilistic atlas‐based mask for comparison with previous work.

To compare this result with our previous work, we examined whether these results were specific to A1. We had previously investigated CS discriminability within a probabilistic definition of Heschl's gyrus (Staib & Bach, 2018), which is likely to contain tissue outside the native‐space A1 definition used here. Hence, we repeated our analysis for a probabilistic group‐level definition of Heschl's gyrus (see white outlines in Figure 4b). This yielded the same main effect of context, although much less pronounced (Table 3). This might indicate that CS encoding is indeed tied to A1, rather than extra‐A1 parts of ACX also contained in Heschl's gyrus. As expected, we did not observe an interaction with complexity, that is, also in the broader definition of HG, CS discriminability was not different between simple and complex sounds.

To examine whether CS/US association was represented in a similar way for both types of sounds, we used cross‐classification and investigated whether CS+/CS− patterns derived from simple sounds also predicted complex CS+/CS− identity, and vice versa. Because initiation of motor responses may influence this result, we compared these values against cross‐prediction of NS, matched for the motor response, as in our previous work. Different from our expectation derived from or previous experiment with frequency sweeps, we did not find that similar patterns distinguished CS+ and CS− for the two types of sounds: Simple CS+/CS− separation was not predicted from complex CS+/CS− separation or the other way around (main effect context: *F*_1, 133_ = 2.32, *p* = .13). Thus, there was no evidence for stimulus‐invariant encoding with the specific types of sounds used here. As we had previously found such stimulus‐invariant encoding particularly in the left hemisphere, we repeated this analysis for the left hemisphere only, but did not find a significant cross‐classification (main effect context within left hemisphere: *F*_1, 57_ = 5.47, *p* = .088). In the right hemisphere, there was no significant cross‐classification either (main effect context within right hemisphere: *F*_1, 57_ < 1, *p* = .86).

### 3.4 Threat representation beyond A1

Finally, we investigated CS discriminability in the superior temporal gyrus excluding A1, using searchlight analysis. This revealed no clusters with a significant effect of context or context × complexity interaction. In a previous study, encoding of the CS/US association was found in a small cluster outside A1. Using this cluster as region of interest, there was no impact of context on classification performance, that is, no evidence for encoding of the CS/US association.

## 4 DISCUSSION

Auditory cortex is required for discriminative fear conditioning with sound CS, but its precise role is unclear until today. In our previous work (Staib & Bach, 2018), we addressed monophone sine sounds and frequency sweeps and suggested that ACX encodes not only (summary statistics of) physical stimulus properties for relay to amygdala, but indeed encodes the CS/US‐association itself. Here, we investigate whether this is restricted to the particular types of sounds standardly used in cross‐species auditory fear conditioning that can be discriminated based on spectro‐temporal features. In contrast to our previous study, the sounds used here are discriminated only by spectral features, that is, multiple frequency components. To allow for a constrained investigation of acoustic complexity, and to distinguish the sound set from the sweeps used in previous studies (Letzkus et al., 2011; Ohl et al., 1999; Staib & Bach, 2018), all temporal features were removed.

Three main findings emerge. First, encoding of a CS/US association for monophone sounds is demonstrated in a region of interest consistent with A1 on a single‐participant level, using a multivariate approach. This finding is replicated but much less pronounced when investigating a probabilistic definition of Heschl's gyrus, containing A1 but also other parts of ACX. This suggests that indeed CS/US association is encoded in, and possibly limited to, A1. A previous finding of CS encoding outside Heschl's gyrus was not replicated here. At the same time, univariate analysis revealed no CS+/CS− differences within our limited brain coverage, which notably excluded a number of areas previously reported in a meta‐analysis of CS+/CS− differences (Fullana et al., 2016). Next, a similar level of CS/US association encoding is seen for musical chords, which are discriminated by spectral features only and do not form part of our natural sound environment. This may suggest that A1 CS/US association encoding is not restricted to sounds that contain temporal discriminative features (Letzkus et al., 2011; Ohl et al., 1999; Weible et al., 2014). We did not directly compare musical chords to the frequency sweeps used in previous cross‐species work (Letzkus et al., 2011; Ohl et al., 1999; Staib & Bach, 2018). However, both in this study on musical chords and in our previous study on frequency sweeps we did not observe different CS/US association encoding for these complex sounds as contrasted with the common comparator, monophone sounds. Nevertheless, the third finding is that the patterns of CS/US association encoding were different between monophone sounds and musical chords, that is, they could not be cross‐decoded. This is different from our previous report (Staib & Bach, 2018) where the patterns distinguishing CS+/CS− were similar for monophone sounds and frequency sweeps and could be cross‐decoded significantly better than between response‐matched NS. As a limitation, sample size and stimulus number for the current study were chosen based on our previous effect size for CS discriminability. In contrast, cross‐decoding performance in this previous study was much lower, and so it is alternatively possible that the current null finding may be a false negative or the previous one a false‐positive. Indeed, we here observed a near‐significant cross‐decoding in the left hemisphere, consistent with particularly pronounced cross‐decoding in the left hemisphere in our previous report. It would be desirable to replicate these findings in a common design using all three kinds of stimuli, and in a larger sample.

It has previously been shown for nondiscriminative fear conditioning with frequency sweeps that A1 relays US information during and shortly after the US occurrence to amygdala, and optogenetic inhibition of this information relay precludes fear conditioning (Letzkus et al., 2011; Weible et al., 2014). While this could suggest a passive role of A1, our present findings speak in favor of an active role in which A1 separately forms US predictions, which may or may not be signaled to amygdala as well. Whether these US predictions are also causally involved in fear learning remains unknown. To investigate the causal role of A1 function during CS presentation, it would be necessary to perturb the formation of these predictions, for example by transcranial magnetic stimulation during CS presentation.

It is possible, however, that the current findings are not specific to fear conditioning. A role of early sensory areas in representing value properties of physical stimuli has become more apparent over the past years, including a representation of reward predictions and reward variance (Bach, Symmonds, Barnes, & Dolan, 2017). The current study cannot disentangle associative learning processes in general from threat conditioning in particular, and this would motivate a direct comparison of threat and reward predictions.

Two alternative interpretations for enhanced discrimination of CS‐evoked BOLD patterns appear unlikely to account for our results. First, ACX activity is known to be influenced by postconditioning receptive field mapping. However, this process is reported to start after about 20 min and to last for several hours (Froemke, Merzenich, & Schreiner, 2007; Schreiner & Polley, 2014), such that we deem it less likely to account for our current results. A second interpretation is top‐down attention. CS+ detection or reinforcement context detection by amygdala may induce resource prioritization (Bach, Hurlemann, & Dolan, 2015), which could increase global auditory attention. Notably, we found no evidence of globally increased BOLD responses in ACX during CS versus NS, or CS+ versus CS− presentation. While A1 receptive fields are thought to be altered by top‐down selective attention (Fritz, David, Radtke‐Schuller, Yin, & Shamma, 2010), and indeed identity of attended‐to‐stimuli can be decoded from BOLD patterns (Riecke et al., 2017), this is usually investigated by presenting competing sounds, whereas in our case, there was no requirement to, and no benefit from, specifically attending to CS. In terms of the task instructions, both contexts required sound identification to the same extent. Furthermore, we previously found that CS‐evoked patterns were similar for monophone sounds and frequency sweeps (Staib & Bach, 2018), and this directly contradicts an underlying mechanism that involves receptive fields. Because there is no reason to expect a difference between the monophone‐CS‐evoked patterns in the previous and current study, this renders an explanation based on selective attention unlikely.

Taken together, we demonstrate auditory CS encoding in an anatomically defined A1 for monophone and musical sounds without temporal discriminative features. This extends a network perspective on fear conditioning (Herry & Johansen, 2014) and motivates future work on the causal role of A1 in this network.

## Data Availability

The data that support the findings of this study are available on request from the corresponding author. The data are not publicly available due to privacy or ethical restrictions.
